# Supplementary material for: Sportsmen’s Attitude towards Dietary Supplements and Nutrition Knowledge: An Investigation in Selected Roman Area Gyms
Source: Nutrients. 2022 Feb 23;14(5):945. doi: 10.3390/nu14050945 (PMC8912719; doi:10.3390/nu14050945)
Supplement: Supplementary file 1 [file nutrients-14-00945-s001.zip › nutrients-1568279-supplementary-done.pdf]

nutrients

MDPI

Supplementary Material

A. The questionnaire

List of the questionnaire items

Section I: Sociodemographic and Lifestyle characteristics (Age, Gender: *M* or *F*, **Weight, Height, BMI, Education***: Bachelor, Graduation, Secondary school, Primary school*; **Alcohol Intake:** *Yes, Not*. **Smoke:** *Yes, Not* **Disease history:** *Yes, Not*. **Job:** *Sedentary, partially sedentary, partially active, Active **)

Section II: Sport activity

How long have you been doing sports? (*Less than 1 month, 1-6 months, 7 months – 1 year, More than 1 year***)**

How many days a week do you train? (*Less than 3 times a week, 3 – 5 times a week, More than 5 times a week*).

On average, how many hours do you train every day? (*Less than 1 hour a day, 1 – 2 hours a day, More than 2 hours a day***).**

Which Type of physical activity do you carry out? (Strength training, Treadmill, Team sports, Water sport, Fighting, and martial arts, Yoga, other).

Section III: Supplement Intake

Do you follow a specific diet? If yes, who recommended it (*Yes, Not*, *if yes indicate who recommended the diet***).**

Do you get any dietary supplements? (*Yes, Not*).

If yes, indicate which supplements (*Amino acids, Creatine, Glutamine, Arginine, Branched-chain amino acids (BCAA), Casein proteins, Whey proteins, Protein powder, Soy proteins, Multivitamins, Mineral salts, B vitamins, Vitamin C, Vitamin E, Carnitine, Protein shakes, Protein bars, Energy drinks, Caffeine, Fish oil pills, Calcium, Iron, Sports bar, Herbal supplements).*

How long have you been getting supplements? **(***More than 2 years, 1 - 2 years, Less 1 year, other***).**

How often do you get supplements? (*Many times a day, One time a day, Weekly, During each workout, Occasionally***).**

For what reason do you use supplements? (*Increase/maintain muscle mass, strength and/or power, Enhance muscular power, Meal, Improve Muscle exercise recovery, Enhance overall athletic performance, Pathologies prevention, prevention of nutritional deficiency, Weight loss, Medical treatments, Enhance immune system, Fat reduction, Stress reduction, Increased alertness, and mental activity, Reduction of post-workout fatigue***).**

Who suggested the use of supplements? (*Medical Doctor, Nutritionist, Instructor/PT/Coach, Internet, Journals, Books, Friends, others***).**

Do you read the label of the supplements? (*Yes, Not).*

Where do you buy supplements? (*Pharmacy, Instructors/coach/PT, Shops specializing in sports nutrition, Online, other***).**

Section IV: nutritional knowledge

Vegetarian athletes should get a daily protein supplement since their needs are not covered by a normal diet (*Yes, Not, I Don't Know***)**

All athletes should take protein preparations as food supplements (*Yes, Not, I Don't Know)*

During an intensive training phase of 60-90 minutes, it is important to take sports drinks (*Yes, Not, I Don't Know***).**

Vitamins B are important for converting food into energy (*Yes, Not, I Don't Know***)**

Carbohydrates are stored as muscle glycogen and are the most important source of energy in sports. (*Yes, Not, I Don't Know***).**

Meal before workout or competition should be rich in proteins. (*Yes, Not, I Don't Know***).**

Fluids deficiency can harm performance. (*Yes, Not, I Don't Know***).**

Taking anabolic steroids for a short time does not have an adverse effect. (*Yes, Not, I Don't Know***).**

Sports drink enhance performance and improve recovery. (*Yes, Not, I Don't Know***).**

To support recovery, an athlete should eat a meal within 1-2 hours after sporting activity. (*Yes, Not, I Don't Know***).**

The amount of sweat lost by an athlete during sports can be determined through weight change before and after the workout. (*Yes, Not, I Don't Know***).**

Athletes' salt assumption should be limited. (*Yes, Not, I Don't Know***).**

Oils derived from plants, fish, nuts, and seeds are considered valuable fats. (*Yes, Not, I Don't Know***).**

Growth, sleep, heart rate, body temperature maintenance, and cell renewal induce energetic (caloric) expenditure. (*Yes, Not, I Don't Know***).**

Creatine is an effective and safe supplement for all ages athletes. (*Yes, Not, I Don't Know***).**

Fruit juices, fruits, vegetables, and soups are the foods that contain the highest quantity of liquids. (*Yes, Not, I Don't Know***).**

White bread contains the same vitamin and mineral quantity as wholemeal bread. (*Yes, Not, I Don't Know***).**

Carbohydrates are the most important nutrient for mental performance. (*Yes, Not, I Don't Know***).**

Weight loss can be due to insufficient food intake, too much physical activity, or both. (*Yes, Not, I Don't Know***).**

Athletes should primarily consume a low-fat diet. (*Yes, Not, I Don't Know***).**

Water is involved in body temperature regulation. (*Yes, Not, I Don't Know***).**

Training without breakfast does not compromise physical and mental performance. (*Yes, Not, I Don't Know***).**

The purity and safety of food supplements are tested before sale. (*Yes, Not, I Don't Know***).**

Fruit juices are an ideal carbohydrate source for training. (*Yes, Not, I Don't Know***).**

When the body gets enough fluids, the urine should be clear or pale yellow. (*Yes, Not, I Don't Know***).**

Proteins are necessary for both the creation and regeneration of muscles. (*Yes, Not, I Don't Know***).**

Iron deficiency may cause fatigue to increase. (*Yes, Not, I Don't Know***).**

Iron is present in meat, green vegetables, eggs, and cereals. (*Yes, Not, I Don't Know***).**

Fat is a crucial source of energy, both at rest and during prolonged exertion. (*Yes, Not, I Don't Know***).**

Performance and recovery are favored by an adequate supply of energy. (*Yes, Not, I Don't Know***).**

Pasta, potatoes, cereals, and bread are high-carb foods. (*Yes, Not, I Don't Know***).**

Multivitamins and minerals always improve performance. (*Yes, Not, I Don't Know***).**

Sodium and potassium are essential electrolytes to obtain good salt and water balance. (*Yes, Not, I Don't Know***).**

**Sedentary:* more than ¾ of the day sitting, *Partially sedentary:* more than ½ of the day sitting, *Partially active:* more than ½ of the day on movement, *Active:* more than ¾ of the day on movement.
